# Factors influencing health professions students’ use of computers for data analysis at three Ugandan public medical schools: a cross-sectional survey

**DOI:** 10.1186/s13104-015-1013-3

**Published:** 2015-02-25

**Authors:** Ian G Munabi, William Buwembo, Francis Bajunirwe, David Lagoro Kitara, Ruberwa Joseph, Kawungezi Peter, Celestino Obua, John Quinn, Erisa S Mwaka

**Affiliations:** Department of Human Anatomy, School of Biomedical Sciences, Makerere University College of Health Sciences, P.O. Box 7072, Kampala, Uganda; Makerere University College of Health Sciences, Kampala, Uganda; Department of Community Health, Mbarara University of Science and Technology, Kampala, Uganda; Department of Surgery, Gulu University, Gulu, Uganda; Department of Pharmacology, School of Biomedical Sciences, Makerere University College of Health Sciences, Kampala, Uganda; Department of Computer Science, College of Computing and Information Sciences Makerere University, Kampala, Uganda

**Keywords:** Computer, Data analysis, Research, Undergraduate, Health profession students

## Abstract

**Background:**

Effective utilization of computers and their applications in medical education and research is of paramount importance to students. The objective of this study was to determine the association between owning a computer and use of computers for research data analysis and the other factors influencing health professions students’ computer use for data analysis.

**Methods:**

We conducted a cross sectional study among undergraduate health professions students at three public universities in Uganda using a self-administered questionnaire. The questionnaire was composed of questions on participant demographics, students’ participation in research, computer ownership, and use of computers for data analysis. Descriptive and inferential statistics (uni-variable and multi- level logistic regression analysis) were used to analyse data. The level of significance was set at 0.05.

**Results:**

Six hundred (600) of 668 questionnaires were completed and returned (response rate 89.8%). A majority of respondents were male (68.8%) and 75.3% reported owning computers. Overall, 63.7% of respondents reported that they had ever done computer based data analysis. The following factors were significant predictors of having ever done computer based data analysis: ownership of a computer (adj. OR 1.80, p = 0.02), recently completed course in statistics (Adj. OR 1.48, p =0.04), and participation in research (Adj. OR 2.64, p <0.01).

**Conclusions:**

Owning a computer, participation in research and undertaking courses in research methods influence undergraduate students’ use of computers for research data analysis. Students are increasingly participating in research, and thus need to have competencies for the successful conduct of research. Medical training institutions should encourage both curricular and extra-curricular efforts to enhance research capacity in line with the modern theories of adult learning.

## Background

Computer use is recognised as an essential requirement of life because of its role in human advancement and learning [1]. This recognition is seen in the need for computer use as a required competence to enhance communication skills, research capacity and information management for students of health professional training curricula [2]. The use of computers for research is on the rise and is facilitated by the increasing availability of: powerful, low cost computers and the additional opportunities for support through collaborative networks using the internet [3]. Computer use for research is enhanced by the availability of appropriate user friendly statistical software. Access to such statistical software is fundamental in the analysis of data by health professions undergraduate students who are often statistically naïve. The ease of understanding statistical and research concepts is directly dependent on the student’s interest and level of exposure to statistical data analysis techniques [4]. This is why hands-on computer use during data analysis training should be a key component of the research methods module in undergraduate and graduate training curriculums [5]. This makes effective utilization of computers and their applications in medical education and research of paramount importance for health professional students [6].

Given the cost of owning a computer by a student, for the institutional planners intending to make computer ownership mandatory, knowing if this choice translates to increased research capacity is important [7]. There are regional collaborations such as Training Health Researchers into Vocational Excellence in East Africa (THRIVE) [8] and Medical Education for Services to All Ugandans (MESAU) – Medical Education Partnership Initiative (MEPI) [9] that aim to develop research capacity in Uganda and East African in general. The MESAU consortium is a country wide partnership of all medical schools in Uganda with John Hopkins University, USA. Research training and support for undergraduate students through such collaborations has a multiplier effect in enhancing research capacity for many generations. It is common for such collaboration to include the purchase of computers for students in their budgets. This applies to both high and low research capacity settings where the alternative uses of computers: use of social media internet sites, watching movies, playing games and so forth, can serve as distractions resulting in a students’ failure to fully exploit the computers power for research.

A study in Nigeria among clinical year medical students reported that 90% of respondents neither had computers nor had routine access to a computer [10]. The authors opined that this may negatively affect the collation and analysis of data as well as the final quality of research projects. Therefore the importance of students owning computers and using them for data analysis cannot be over emphasized particularly in low resource settings like ours. In this study we set out to determine the association between owning a computer and use of computers for research data analysis and the other factors influencing health professional student computer use for data analysis within the MESAU- MEPI consortium. This will in turn be instrumental in increasing research capacity in medical training institutions in Uganda.

## Methods

This was a cross sectional study conducted on undergraduate health professions students at the three public universities in the MESAU-MEPI consortium. The participating schools included Makerere University College of Health Sciences (MakCHS), Mbarara University of Science and Technology (MUST) and Gulu University (GU). The total number of eligible students was 2772 (MakCHS 1372, MUST- 1050, GU- 350). Each of the three universities in the study has its own different academic curriculum, thus the timing, duration of teaching and content of the biostatistics, epidemiology and research methods course is not harmonized across the three institutions. However, students receive an introductory course on biostatistics and research methods during their first year of study. In their third and fourth years they learn project proposal development and implementation, and they are also required to undertake research projects and write reports.

The survey included undergraduate student respondents pursuing various health profession degree programs that included: Nursing, Pharmacy, Cytotechnology, Biomedical Sciences, Medical radiography, Dentistry, and Medicine and Surgery.

The target sample size of 668 respondents for the study was calculated assuming a 50% prevalence for the outcome factor, precision of 4%, power of 99%, and design effect of 1.2 to cater for clustering of the sample population in the three universities. We used the online calculator for sample size using the single proportion and available on the openepi web site [11].

### Questionnaire

The questionnaire was adapted from three sources. First, questions on undergraduate teaching in research methods and biostatistics were adapted from a questionnaire that was developed for faculty in the University of East Anglia to provide user related recommendations for the development of statistical education by the General Medical Council of UK *tomorrows doctors* [12-14]. *Tomorrows doctors*, is a document that is used to guide and ensure quality in provision of health professional training in the United Kingdom [13-15]. Second, questions on attitudes and perceptions of biostatistics and research methods were adapted from a questionnaire developed by the Dow University of Health Sciences, Karachi, Pakistan [16]. To these we added questions on research participation and computer use for research. The questionnaire was initially piloted on 15 students, and highlighted ambiguities were corrected in the final version. Pilot questionnaires were excluded from the final analysis. This paper focuses on research participation and computer use for research.

### Data collection and analysis

Class representatives were recruited as research assistants to administer and collect the duly completed questionnaires. The class representative used convenience sampling to select the respondents. Questionnaires were distributed at the end of lectures; they were placed at the front of the class and interested students were requested to pick a copy after the lecture. The data from the questionnaires was captured using Epidata (Epidata association, Denmark) and exported to STATA 12 (StataCorp LP, Texas, USA) for analysis.

The results of analysis were summarized using frequencies, means and standard deviations. Inferential statistics were obtained using univariable and multi-level logistic regression to cater for the clustering of respondents at each university. All variables were included in the initial model. Female gender was retained in the final model due to previously reported significant associations with computer based data analysis [17,18]. Backward multi-level logistic regression using the gllamm function in STATA was used to calculate the odds ratios with iterations nipped at 60 for final modeling [19]. The level of significance was set at 0.05, using computer based data analysis as the dependent variable. Observations with missing values were dropped from analysis on running the various tests.

Ethical approval was obtained from the School of Biomedical Sciences Research (IRB number SBS-045) and registration done with the Uganda National Council of Science and Technology. Informed consent was obtained from all participants before enrolment into the study. No personal identifier details were captured or used at any part of the analysis. Students received a drink and small snack equivalent to 1 US dollar as refreshment for participating in the study. Refusal to participate in the study did not in any way affect the student’s access to materials or services they were entitled to.

## Results

### Baseline characteristics

Six hundred and sixty eight (668) questionnaires were distributed and of these 600 were completed and returned giving an overall response rate of 89.8%. Response rates per site were: MakCHS, 440/452 (97.3%), MUST, 127/150 (84.7%), and GU, 33/66 (50%). Overall 600/2772 (21.6%) of the student population participated in the study. Table 1 shows the summary of the descriptive statistics according to the respondent’s university. A majority of respondents were in their third and fourth years of study. The data show that 63.7% of respondents reported having ever participated in any form of research. About 75.3% of respondents reported that they owned computers and 50.3% reported that they had ever done computer based data analysis.

**Table 1:** **Descriptive statistics of participants according to University**

**University**	**MakCHS**	**MUST**	**GU**	**Overall**
Gender (n, % female)	342 (29.8)	66 (39.4)	18 (22)	426* (31.2)
Computer ownership (n, % yes)	437 (74)	124 (79)	29 (86.1)	591* (75.3)
Computer use for data analysis (n, % yes)	440 (40)	107 (96.3)	30 (20)	577 (50.3)
Participated in research (n, % yes)	436 (67)	124 (47.6)	31 (83.9)	591 (63.7)
Year of study (mode)	4	3	4	3
Were research methods taught (mode)^1^	1	1	1	1
Were methods taught useful (mode)^1^	1	1	1	1
Total (n, % total)	439 (73.3)	127 (21.2)	33 (5.51)	599^2^

^1^items coded as 0 = “No”, 1 = “Yes” and 2 = “I don’t recall.

^2^Note one respondent did not indicate the university they were from.

*Missing data.

### Predictors of computer based data analysis

Although female respondents were less likely to report having done computer based data analysis than the male respondents, this was not significant (OR 0.93, 95% CI 0.62 to 1.42, p = 0.75) (Table 2). Significant variables on univariable analysis for computer based data analysis were: owning a computer (OR 1.68, p <0.01) and having had statistics taught in a recently completed course (OR 1.71, p <0.01). There was no significant difference in the responses from respondents that reported having ever participated in any research compared with those who had not participated in any research (OR 1.32, p = 0.11). MUST had the youngest respondents (p <0.01) and also had the lowest number of respondents that reported ever participating in research (p <0.01). GU had the lowest number of respondents that reported having ever done computer based data analysis (p <0.01). MakCHS had the lowest number of students that could recall having been taught statistics in their most recently concluded course (p <0.01). The odds for use of computers in data analysis increased significantly with respect to the university the respondent was from (P <0.01).

**Table 2:** **Logistic regression analysis to determine the factors associated with computer based data analysis**

**Variable**	**OR**	**95% CI**	**P value**	**Adj. OR**	**95% CI**	**P value**
Institution						
MakerereUniversity^1^	1.0			-		
Gulu	0.36	0.14- 0.90	0.03			
Mbarara	37.24	13.47- 102.98	<0.01			
Gender	0.93	0.62-1.41	0.75	0.76	0.46-1.23	0.26
Year of study	0.91	0.79-1.05	0.21	-		
Participated in research	1.32	0.94-1.87	0.11	**2.64**	**1.62-4.33**	**<0.01**
Owned a computer	1.68	1.14-2.48	<0.01	**1.80**	**1.12-2.89**	**0.02**
Were research methods taught?	1.71	1.25-2.34	<0.01	**1.48**	**1.02-2.14**	**0.04**
Were the methods taught useful?	1.59	0.84-3.00	0.15	-		

^1^This was coded as Makerere “0”, Mbarara “1” and Gulu “2”.

Items in bold remained significant after adjustment (p < 0.05).

The final model had the following variables: the respondents’ gender, participation in research, owning a computer and ‘where research methods recently taught?’ Participation in research was associated with an almost 3 fold increase in use of computers for data analysis (adj.OR 2.64, P < 0.01). Owning a computer was associated with an 80% increase in odds of a respondent having done computer based data analysis. Finally having recently been taught statistics was associated with a 48% increase in the odds of a student doing computer based data analysis keeping the other variables in the model constant (Table 2).

## Discussion

We set out to determine the association between owning a computer and use of computers for data analysis and the other factors influencing health professional student computer use for data analysis within the MESAU- MEPI consortium. We found that students who reported having done computer based data analysis were 80% more likely to own a computer than those that had not (p = 0.02). Also the students who had done computer based data analysis were more like to have participated in research (p < 0.01) or had recently completed a course where research methods were taught (p = 0.04). Modern theories of adult learning, in particular the behavioural and experiential theories are relevant to the above observations. Behavioural theories emphasize educator- centred instruction and the learning of facts and skills that educators have decided are important. They also involve the learning of the skills and information in small units, and the provision of feedback and reinforcement to students. Experiential theories essentially focus on developing competencies and practicing of acquired skills. Educators are responsible for creating, facilitating access to and organizing experiences in order to facilitate learning [20]. In relation to the above theories one can argue that computer based data analysis as an activity or behaviour is a product of the student’s deeper appreciation of the computers role in research [20]. There are many applicable implications, of this link between the findings from this study and adult theories of learning, for health professional training institutions. For example an institution whose mission and vision place emphasis on electronic research communication (publications, blogs, wikis etc.) will work to ensure that their students learning experiences include publication according to the experiential learning theoretical framework [20]. Teachers will be expected to model or demonstrate a certain level of expertise in use of various applications to facilitate the students learning [20,21]. Finally the assessment needs to be aligned to ensure that the institutional goals as articulated in the mission and vision statements are delivered by the curriculum [20]. This makes this topic very important to all stakeholders especially as students are increasingly participating in research as an academic requirement; as part of faculty-student collaboration [22]; and as small grants funded projects [23]. In this era of evidence based medicine it is thus imperative that students acquire knowledge and skills relevant to their education and future practice [24].

At the other extreme of the spectrum we observed that although three quarters of students owned computers not all of them had ever done computer based data analysis. Whereas MakCHS contributed the majority of participants it had the lowest number of students that had ever used computers for analysis as compared to MUST. The reasons for this might arise from the fact that MUST is located in the country-side, and historically has a stronger community health component than MakCHS. MUST being a smaller University, has a higher level of faculty-student interaction in contrast to MakCHS where the large student to faculty ratio leads to use of didactic methods of instructions, a reduced emphasis on hands-on approaches to teaching research methods, reduced student teacher interaction and student mentoring [22]. Also, many of the students who reported participating in research had never done computer based data analysis. This represents a missed opportunity on the part of the participating institutions. For students to perform computer based data analysis they need to have some basic knowledge and competence in using statistical software [25]. Institutions of higher learning are increasingly expected to demonstrate that their graduate students indeed have such competencies [2]. Recently one of the Ugandan Universities made computer ownership a requirement for all students [7]. It is important to note that computer ownership does not guarantee its usage for research purposes as has been demonstrated in this study. Whereas computer based data analysis is not explicitly taught to undergraduates in Ugandan medical schools there exist curricula opportunities for student experiential learning of these concepts through self-learning, and from peers, post-graduate students and mentors. A failure to use these curricula opportunities may explain the above computer use for data analysis observations and previously reported observations from undergraduate students not participating in research because of time constraints, inadequate knowledge and lack of guidance, collaboration and funding [26]. Other factors that have been cited as barriers to undergraduate interest in research are: the lack of opportunities to carry out research; the lack of awareness of potential research projects; and the lack of interest from potential supervisors [27,28]. There is a possibility that some students do not take the research component of their training seriously. This can all change with institution wide interventions that result in a new trend in which computer ownership translates into enhanced research capacity [20,22].

Some of the documented efforts at getting student computer ownership to translate into increased research capacity include: (1) the formation of a ‘Research and Writers’ club’ that aims to nurture and cultivate a culture of research and scientific writing among undergraduate students [29]; (2) promoting participation in research which as observed in this study was associated with an almost 3 fold increase in use of computers for data analysis. The MESAU consortium has increased research opportunities for students by offering incentives and support for them to undertake locally relevant research projects with an aim of enhancing their research capacity [23]; (3) selection of an institution preferred statistical computing software and the requirement that students should use increasingly complex forms of statistical approaches as they mature to become professionals. Use of a single institution wide preferred statistical computing software enhances the students’ understanding of these concepts by coupling all aspects of the curriculum with hands-on use. Using single institution wide preferred statistical computing software also promotes students’ familiarity with exposure to increasingly complex statistical routines while saving time by using a single statistical computing working/learning environment/context across the institution [30-32]; and, (4) it is important to note that students do not have to learn the principles of research methods from the lecture rooms alone, but, also from faculty by observation and working together on research projects and publications. It is therefore essential that students are continuously engaged in research activities by their mentors. Such a diversity of instructional delivery methods allows students to learn and appreciate the depth and breadth of research applications as they progress from basic sciences to clinical disciplines [33]. If this is reflected in the assessment process of the institution then the fact that “student assessment drives learning” can be used to guarantee that this competence of information literacy is attained while making research exciting and relevant to the student [32,34]. This justifies the current practice in some of these universities where to foster computer based data analysis students and faculty are encouraged to do analysis on their own by discouraging the payment of statisticians as a budget item for small grants and projects leading to the award of a degree.

Our study had two limitations that need to be considered. The use of a self-administered questionnaire and convenient sampling expose results to potential bias. Second, some respondents chose not to answer all questions in the survey; however we feel that this effect is negligible. The study also had several strengths. First, the study had a very good overall response rate of 89.8%. Second, three out of five medical schools in Uganda participated in this study. MakCHS and MUST are the oldest and biggest medical schools in Uganda and together they account for almost two-thirds of medical students in the country. Therefore our results are likely to be representative of most academic medical institutions in the country. Third, anonymity of questionnaires reduced social desirability bias where respondents portray themselves positively. Fourth, the study had broad representation; it included students from different health professional programs.

Much of the teaching of statistics and research methodology is focused on refining the cognitive aspects (such as aptitudes and knowledge) students are expected to develop while ignoring the non-cognitive aspects (such as outlooks, perspectives, expectations and inspirations) [35]. Therefore a study on these non-cognitive aspects should be done for a more valid assessment of the factors that influence students’ utilization of computer for data analysis to be done.

## Conclusions

Owning a computer, participation in research and undertaking courses in research methods influence undergraduate students’ use of computers for research data analysis. Students are increasingly participating in research, and thus need to have competencies for the successful conducting of research. Since computer ownership does not guarantee its usage for research purposes, health professional training institutions should encourage both curricular and extra-curricular efforts to enhance research capacity in line with the modern theories of adult learning.

## Abbreviations

GU: Gulu University
MakCHS: Makerere College of Health Sciences
MEPI: Medical education partnership initiative
MESAU: Medical Education for Services to All Ugandans
MUST: Mbarara University of Science and Technology
THRIVE: Training Health Researchers into Vocational Excellence in East Africa
